# Characterization of HIV-1 *gag* and *nef* in Cameroon: further evidence of extreme diversity at the origin of the HIV-1 group M epidemic

**DOI:** 10.1186/1743-422X-10-29

**Published:** 2013-01-22

**Authors:** Marcel Tongo, Darren P Martin, Lycias Zembe, Eitel Mpoudi-Ngole, Carolyn Williamson, Wendy A Burgers

**Affiliations:** 1 Division of Medical Virology, University of Cape Town, Cape Town, South Africa; 2 Institute of Medical Research and Study of Medicinal Plants, Yaoundé, Cameroon; 3 Computational Biology Group, Institute of Infectious Diseases and Molecular Medicine, University of Cape Town, Cape Town, South Africa; 4 National Health Laboratory Service, Groote Schuur Hospital, Cape Town, South Africa

**Keywords:** HIV-1 diversity, West central Africa, RDP3, Maximum likelihood, PHYML

## Abstract

**Background:**

Cameroon, in west central Africa, has an extraordinary degree of HIV diversity, presenting a major challenge for the development of an effective HIV vaccine. Given the continuing need to closely monitor the emergence of new HIV variants in the country, we analyzed HIV-1 genetic diversity in 59 plasma samples from HIV-infected Cameroonian blood donors. Full length HIV *gag* and *nef* sequences were generated and phylogenetic analyses were performed.

**Findings:**

All *gag* and *nef* sequences clustered within HIV-1M. Circulating recombinant form CRF02_AG predominated, accounting for 50% of the studied infections, followed by clade G (11%), clade D and CRF37_cpx (4% each), and clades A, F, CRF01_AE and CRF36_cpx (2% each). In addition, 22% of the studied viruses apparently had *nef* and *gag* genes from viruses belonging to different clades, with the majority (8/10) having either a *nef* or *gag* gene derived from CRF02_AG. Interestingly, five *gag* sequences (10%) and three (5%) *nef* sequences were neither obviously recombinant nor easily classifiable into any of the known HIV-1M clades.

**Conclusion:**

This suggests the widespread existence of highly divergent HIV lineages in Cameroon. While the genetic complexity of the Cameroonian HIV-1 epidemic has potentially serious implications for the design of biomedical interventions, detailed analyses of divergent Cameroonian HIV-1M lineages could be crucial for dissecting the earliest evolutionary steps in the emergence of HIV-1M.

## Findings

The Congo basin in west central Africa is thought to be the origin of HIV, where several cross-species transmission events from chimpanzees to humans occurred [1,2]. Cameroon, located in this region, has one of the most genetically diverse HIV epidemics in the world [3-6]. Alongside CRF02_AG, which accounts for more than half of infections in the country, circulating virus lineages include every known HIV-1M subtype, numerous circulating recombinant forms (CRFs) and a variety of apparently unique recombinant forms (URFs) [7]. The prevalence of HIV-1 in Cameroon is one of the highest in west central Africa, at 5.3% [8]. This, together with the co-circulation of divergent variants of multiple clades, has created the conditions for frequent mixed infections and inter-clade recombination. Constantly improving phylogenetics-based analytical techniques and rapidly expanding HIV sequence datasets allow for better characterization of diverse sequences, and promise to yield important insights into the origin, evolution and spread of HIV-1.

Given the potential impact of HIV-1 diversity on both vaccine development and the sustainability of antiretroviral therapies, it is particularly important that molecular epidemiological surveillance is continued in HIV diversity hotspots such as Cameroon. In this study we have focused on characterizing the diversity of *gag* and *nef* genes of Cameroonian HIV-1 isolates. These genes are particularly relevant because they encode highly immunogenic proteins that are frequently included in candidate vaccines [9-11]. We sequenced 50 full length HIV-1 *gag* and 55 *nef* genes from 59 HIV-infected blood donors in Cameroon. To obtain a phylogenetic view of Cameroonian HIV diversity that explicitly accounted for the confounding effects of recombination, we performed extensive recombination-aware phylogenetic analyses of these new sequences along with publically available homologous HIV-1M *gag* and *nef* sequences from the Congo basin and a representative selection of the major known HIV lineages from the rest of the world. These representative sequences were selected to include the broadest diversity of sequences previously identified as belonging to these known clades by constructing maximum likelihood trees from all available *gag* and *nef* sequences for each clade, and selecting one sequence from each of the up to ten most basal lineages from the root of these clades.

Anonymously-donated HIV-infected blood units were collected between December 2006 and August 2007 from Yaoundé Central Hospital, Cameroon, in a study approved by the National Ethics Committee of the Cameroonian Ministry of Health and the University of Cape Town. Although no data on risk factors for HIV was available for the blood donors, they are believed to represent the general adult population of Yaoundé. All donors were antiretroviral therapy naïve and only age and gender information were obtained.

RNA was extracted from plasma samples, reverse transcribed and PCR amplified as described previously [12] using subtype non-specific HIV-1 primers for HIV-1 full length *gag *[12] and *nef *[13] genes, and sequenced. Sequenced fragments were assembled using ChromasPro. Full length *gag* and *nef* sequences were generated and aligned using MUSCLE with manual editing in MEGA5, together with a representative selection of 270 *gag* and 279 *nef* HIV sequences from the rest of the world and all other published *gag* (266) and *nef* (278) sequences from Cameroon and other west central African countries available in the LANL (http://www.hiv.lanl.gov/content/sequence/HIV/mainpage.html) and Genbank databases. Maximum likelihood phylogenetic trees were constructed from these sequences with 100 full maximum likelihood bootstrap replicates (implemented in PHYML [14]), following either complete removal of recombinant sequence fragments or the division of recombinant sequences into their constituent fragments by a blinded fully exploratory screen for recombination using RDP3 [15]. The recombination screen was fully exploratory in that every sequence was analysed for evidence of both intra- and inter-clade recombination. Either full *nef* and *gag* sequences or the sub-fragments of these sequences identified as having recombinant origins were classified as belonging to particular HIV clades if they clustered with reference sequences of these clades. Divergent sequences were defined as those residing on isolated branches outside of subtrees containing previously defined HIV-1 subtype or CRF lineages. Outlier sequences on the other hand were defined as those residing on basal branches of subtrees containing previously defined HIV-1 subtype or CRF lineages. Nucleotide sequences were deposited in GenBank [JX244899-JX244948 for *gag* and JX244949-JX245003 for *nef*.

Clinical and demographic data of the HIV-infected Cameroonian study participants are summarized in Table 1. Individuals had a median age of 31 years and the majority were male. They had a median CD4 count of 432 cells/mm^3^ and a median viral load of approximately 100 000 RNA copies/ml.

**Table 1:** Characteristics of study individuals (n=59)

	
Age^a^ (years)	31 (19–54)
Sex (Male: Female)	46: 13
CD4 count^a^ (cells/mm^3^)	432 (42–1972)
Viral load^a^ (Log RNA copies/ml)	4.93 (3.29-6.32)

^a^Median and range.

**Figure 1:**
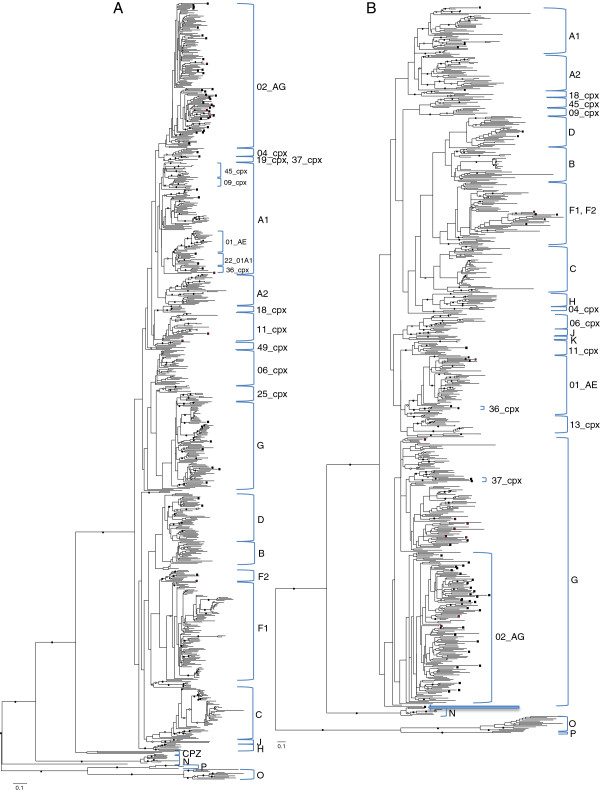
**Maximum likelihood trees indicating the phylogenetic relationships between 727 *gag *(A) and 628 *nef *(B) sequences of HIV-1.** The trees were constructed from these sequences with 100 boostrap replicates following removal of recombinant sequence fragments by a blinded fully exploratory screen for recombination using RDP3. Black squares at the end of the branches represent the *gag* and *nef* sequences sampled from Cameroon in this study, while red squares represent intragene recombinant fragments in our samples. The *gag* tree was rooted using HIV-1 group N, O, P and SIV CPZ isolates, while the *nef* tree was rooted with HIV-1 group N, O and P isolates. Solid and open circles indicate branches with greater than 70% and 50% bootstrap support, respectively. The arrow in the *nef* tree indicates an outlier of both clades G and CRF02_AG.

**Figure 2:**
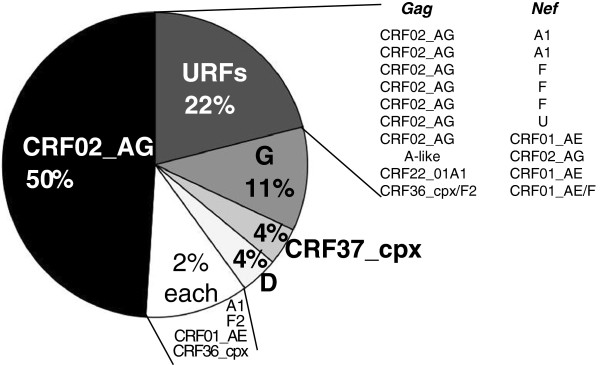
**Pie chart summarizing the distribution of HIV-1 Group M clades and recombinant forms from full length *gag *and *nef *gene sequencing (n=46).** Intergene recombinants are detailed in the right panel. The 13 samples that were typed in only one of the genes were excluded from this analysis.

All our sequences were derived from HIV-1 group M viruses (Figure 1 and Additional files 1, 2). The sequences clustered with different clades and circulating recombinant forms distributed throughout the phylogenetic trees (Table 2), consistent with the breadth of HIV-1 diversity previously described in Cameroon. CRF02_AG-like viruses dominated the clade distribution, infecting 50% of the 46 participants for which both genes were sequenced (Figure 2). Participants infected with viruses having both *nef* and *gag* clustering within known HIV-1M clades included those belonging to clades G, D, A, and F. Subtype G sequences accounted for 11% of infections, subtype D for 4% and sub-subtypes A1 and F2 for 2% each. In addition to CRF02_AG, other CRFs identified were CRF37_cpx (4%), and CRF01_AE and CRF36_cpx (2% each). Additionally, in two samples for which only *gag* or *nef* was typed, these were classified as belonging to CRF11_cpx. Notably, despite subtypes B and C collectively accounting for approximately 75% infections worldwide [16], none of our sequences were classified as belonging to either of these clades. 

**Table 2:** Clade distribution of HIV-1 in Cameroon

**Sample ID**	***gag*gene**	***nef*gene**	**Genotype**
BS01	CRF02_AG	CRF02_AG	**CRF02_AG**
BS02	A-like^b^	CRF02_AG	**URF**
BS03	G	G	**G**
BS04	G	ND^c^	**G**^**a**^
BS05	CRF02_AG	ND^c^	**CRF02_AG**^**a**^
BS06	CRF02_AG	CRF02_AG	**CRF02_AG**
BS09	CRF02_AG	A1	**URF**
BS10	A1	A1	**A1**
BS11	CRF02_AG	F	**URF**
BS12	G	G	**G**
BS13	CRF02_AG	A1	**URF**
BS14	CRF02_AG	CRF02_AG	**CRF02_AG**
BS16	CRF02_AG	CRF02_AG	**CRF02_AG**
BS18	ND^c^	CRF02_AG	**CRF02_AG**^**a**^
BS19	CRF02_AG	CRF02_AG	**CRF02_AG**
BS20	ND^c^	CRF02_AG	**CRF02_AG**^**a**^
BS21	CRF02_AG	CRF02_AG	**CRF02_AG**
BS22	CRF02_AG	CRF02_AG	**CRF02_AG**
BS23	CRF02_AG	CRF02_AG	**CRF02_AG**
BS24	CRF37_cpx	CRF37_cpx	**CRF37_cpx**
BS25	CRF02_AG	F	**URF**
BS26	CRF01_AE^b^	CRF01_AE	**CRF01_AE**
BS27	CRF37_cpx^b^	CRF37_cpx	**CRF37_cpx**
BS29	CRF02_AG	U^b^	**URF**
BS30	D	D	**D**
BS31	ND^c^	CRF02_AG	**CRF02_AG**^**a**^
BS32	CRF02_AG	CRF02_AG	**CRF02_AG**
BS35	ND^c^	CRF11_cpx	**CRF11_cpx**^**a**^
BS38	CRF02_AG	CRF02_AG	**CRF02_AG**
BS39	CRF02_AG	CRF02_AG	**CRF02_AG**
BS40	CRF36_cpx	CRF36_cpx	**CRF36_cpx**
BS42	URF	CRF01_AE^b^	**URF**
BS43	CRF02_AG	CRF02_AG	**CRF02_AG**
BS44	ND^c^	CRF02_AG	**CRF02_AG**^**a**^
BS45	CRF02_AG	CRF02_AG	**CRF02_AG**
BS46	G	G	**G**
BS47	CRF02_AG	CRF02_AG	**CRF02_AG**
BS48	G	G	**G**
BS49	F2	F	**F2**
BS50	CRF02_AG	CRF02_AG	**CRF02_AG**
BS51	G	G	**G**
BS53	CRF02_AG	CRF02_AG	**CRF02_AG**
BS54	D	D	**D**
BS55	CRF02_AG	F	**URF**
BS56	CRF02_AG	CRF02_AG	**CRF02_AG**
BS57	CRF11_cpx^b^	ND^c^	**CRF11_cpx**^**a**^
BS64	CRF02_AG	CRF02_AG	**CRF02_AG**
BS65	CRF22_01A1	CRF01_AE	**URF**
BS66	CRF02_AG	CRF02_AG	**CRF02_AG**
BS71	CRF02_AG	CRF02_AG	**CRF02_AG**
BS72	CRF36_cpx^b^/F2^b^	CRF01/F	**URF**
BS73	CRF02_AG	CRF02_AG	**CRF02_AG**
BS74	ND^c^	A-like^b^	**A-like**^**a**^
BS75	CRF02_AG	CRF02_AG	**CRF02_AG**
BS77	CRF02_AG	CRF02_AG	**CRF02_AG**
BS78	ND^c^	CRF01_AE	**CRF01_AE**^**a**^
BS79	ND^c^	CRF02_AG	**CRF02_AG**^**a**^
BS81	CRF02_AG	ND^c^	**CRF02_AG**^**a**^
BS82	ND^c^	CRF02_AG	**CRF02_AG**^**a**^

^a^Genotype was uncertain in samples for which only a single gene was determined.

^b^Outlier; ^c^ND: Not done.

In 10/46 samples from which both *nef* and *gag* sequences were analysed, they were classified as belonging to different clades from one another. One of the two gene sequences from 8/10 of these patients were classified as CRF02_AG (Figure 2 and Table 3). In addition, we detected numerous recombination breakpoints within the genome regions analysed. Among the newly sequenced *gag* genes, three (6%) were identified as containing recombination breakpoints between gene segments that phylogenetically clustered within two distinct groups of the same clade, indicating that they were likely intra-clade recombinants. Whereas two of these (BS05 and BS55) were CRF02_AG/02_AG recombinants, one (BS57) was a CRF11_cpx/11_cpx recombinant (Table 3). Four of the newly sequenced *nef* genes (7%) also showed evidence of intra-clade recombination, including three (BS12, BS48 and BS51) which were intra-subtype G recombinants and one (BS39) which was an intra-CRF02_AG recombinant. Whereas one of the newly sequenced *gag* genes (BS72) was apparently derived through recombination between F2 and CRF36_cpx parental viruses, one of the *nef* genes was apparently derived through recombination between F and CRF22_01A1 parental viruses.

**Table 3:** Inter and intraclade recombinants

**Sample ID**	***gag*gene**	***nef*gene**
BS02	A-like^a^	CRF02_AG
BS09	CRF02_AG	A1
BS11	CRF02_AG	F
BS13	CRF02_AG	A1
BS25	CRF02_AG	F
BS29	CRF02_AG	U^a^
BS42	CRF02_AG	CRF01_AE^a^
BS39	CRF02_AG	CRF02_AG/CRF02_AG
BS55	CRF02_AG/CRF02_AG	F
BS05	CRF02_AG/CRF02_AG	ND^b^
BS65	CRF22_01A1	CRF01_AE
BS72	CRF36_cpx^a^/F2^a^	CRF01_AE/F
BS57	CRF11_cpx^a^/CRF11_cpx	ND^b^
BS12	G	G/G
BS48	G	G/G
BS51	G	G/G

^a^Outlier; ^b^ND: Not done.

The phylogenetic analysis of *gag* sequences derived from the Cameroonian samples further revealed four sequences (BS09, BS25, BS16 and BS42) situated on divergent branches near the base of the CRF02_AG subtree, highlighting the remarkable diversity within the CRF02_AG clade in Cameroon (indicated by blue squares in Additional file 1). Furthermore, five other Cameroonian *gag* sequences determined here branched very near the base of the clades that they grouped with: BS02 from the base of CRF09_cpx/CRF45_cpx, BS27 from the base of CRF37_cpx, BS26 from the base of CRF01_AE, BS57 from the base of the CRF11_cpx and BS72 from the base of the F2 and CRF36_cpx clades (blue arrows in Additional file 1). Similarly, in *nef*, three such sequences also branched from near the base of the clades that they were most closely clustered with: BS74 near the base of the A clade, BS42 near the base of the CRF01_AE clade and BS29 near the base of the CRF02_AG/G clades (blue arrows in Additional file 2).

Several studies have characterized HIV-1M sequences from Cameroon [3-7]. Our analysis of all available full-length *gag* and *nef* gene sequences from west central Africa clearly reinforces the findings of these previous studies regarding the high degree of HIV-1M genetic diversity in this country. Also, unsurprising in the light of previous Cameroonian diversity studies, was our finding that most of the newly sampled sequences are likely CRF02_AG (accounting for 50% of HIV-1M infections), with the other “pure” subtypes (G, D, A, and F) and CRFs (CRF11_cpx, 36_cpx, 37_cpx, and CRF01_AE) accounting for the remainder of infections.

CRF02_AG and clade G viruses are broadly distributed across west central Africa and have apparently been circulating stably there for many years [3,17-19], consistent with the presence of fragments of these viruses having been identified in a large number of CRFs and URFs that have been sampled from this region. Our analysis demonstrated that these two clades are highly diverse, and in most instances where *gag* and *nef* sequences from an individual patient had discordant clade classifications, the sequence of one of the genes clustered within the CRF02_AG clade, reinforcing the notion that this viral clade is a major contributor of genetic material to new recombinants [20]; an alternative explanation, however, could be that the *gag* and *nef* genes were amplified from different viruses co-infecting the same patients. Ongoing molecular and clinical surveillance will reveal whether new recombinants will begin to circulate stably, will harbor biological properties that favor their transmission, or will impact clinical outcomes.

Carr et al. [7] recently identified sequences that were outliers of various HIV-1M clades, and presented analyses that many of these viruses were likely URFs, which might explain the phylogenetic placement of these sequences on the outskirts of known clades. Although the majority of the outlier viruses found in our study were also URFs, they remained outliers after the removal of recombinant segments. It thus appears that these sequences represent viruses that are genuinely highly divergent and are possibly extant descendants of previously unknown early diverging HIV-1M lineages. Such sequences could help tremendously with efforts to piece together the early evolutionary history of HIV-1M. For example, sequences such as BS29, which are outliers of both the CRF02_AG and G clades may help to resolve the controversy surrounding the origin of CRF02_AG [20-22].

In summary, our data show the predominance in an urban Cameroonian setting of HIV-1 CRF02_AG viruses alongside viruses belonging to known HIV-1M clades, URFs and currently unclassified divergent lineages. We are currently performing full-genome sequencing to further characterize the divergent sequences identified here.

## Abbreviations

HIV: Human Immunodeficiency Virus; CRF: Circulating recombinant form; URF: Unique recombinant form; RNA: Ribonucleic acid; PCR: Polymerase Chain Reaction.

## Competing interests

The authors declare that they have no competing interests.

## Authors’ contributions

MT carried out the laboratory work and phylogenetic analyses, with assistance from LZ and DM. CW and EMN conceived of the study, and participated in its design and coordination. WB and DM supervised the work. MT, DM and WB wrote the manuscript. All authors read and approved the final manuscript.

## Supplementary Material

Additional file 1 **Detailed phylogenetic analysis of nucleotide sequences in the *gag *gene.** Maximum likelihood tree indicating the phylogenetic relationships between 727 *gag* sequences including all sequence identifiers. Blue arrows indicate the outlier sequences found in this study while the green arrows indicate the outlier sequences from previous Cameroonian sequences. Black squares at the end of the branches represent the *gag* sequences sampled from Cameroon in this study, while red squares represent intragene recombinant fragments in our samples. The blue squares show the new divergent branches formed by viruses sampled in this study. Sequence C.ZM.2006.ZM1464F appears to have been mis-labelled in the LANL database, and consistently groups with subtype A1. Click here for file

Additional file 2 **Detailed phylogenetic analysis of nucleotide sequences in the *nef *gene.** Maximum likelihood tree indicating the phylogenetic relationships between 628 *nef* sequences including all sequence identifiers. Blue arrows indicate the outlier sequences found in this study while the green arrows indicate the outlier sequences from previously-characterized Cameroonian sequences. Black squares at the end of the branches represent the *nef* sequences sampled from Cameroon in this study, while red squares represent intragene recombinant fragments in our samples. Click here for file
