# Supplementary material for: Characterization of HIV-1 gag and nef in Cameroon: further evidence of extreme diversity at the origin of the HIV-1 group M epidemic
Source: Virol J. 2013 Jan 22;10:29. doi: 10.1186/1743-422X-10-29 (PMC3560183; doi:10.1186/1743-422X-10-29)
Supplement: Additional file 1 — Detailed phylogenetic analysis of nucleotide sequences in the gag gene. Maximum likelihood tree indicating the phylogenetic relationships between 727 gag sequences including all sequence identifiers. Blue arrows indicate the outlier sequences found in this study while the green arrows indicate the outlier sequences from previous Cameroonian sequences. Black squares at the end of the branches represent the gag sequences sampled from Cameroon in this study, while red squares represent intragene recombinant fragments in our samples. The blue squares show the new divergent branches formed by viruses sampled in this study. Sequence C.ZM.2006.ZM1464F appears to have been mis-labelled in the LANL database, and consistently groups with subtype A1. [file 1743-422X-10-29-S1.pptx]

## Slide 1
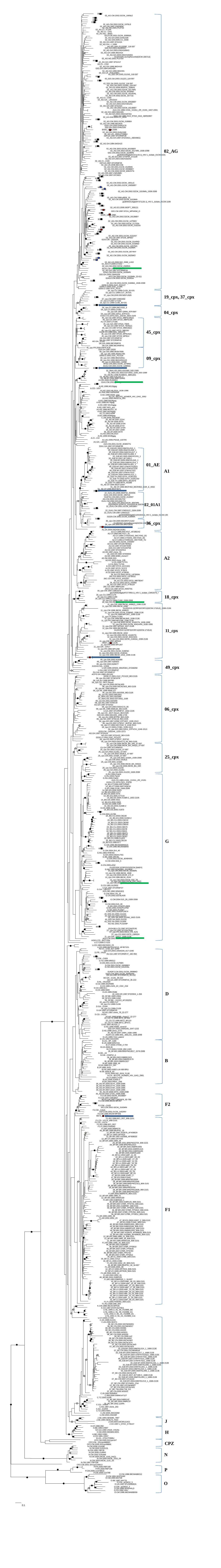

BS29
02_AG.CM.2002.02CM_1669LE
BS01
BS53
BS81
02_AG.CM.2002.02CM_1970LE
02_AG.CM.1997.CM53658
02_AG.NG.2001.PL0710
02_AG.CI.-.IC144
02_AG.CI.-.CI51
02_AG.NG.-.IBNG
0213.CM.2001.01CM_0096MA
02_AG.GH.2003.GHNJ196
02_AG.GW.2005.CC_0048
02_AG.GH.1997.97GH24
02_AG.GH.-.I_2496
AG.NG.2001.PL0209P_118-597
02G.GH.2003.AB480047
02_AG.GH.2003.03GH189AG
02_AG.GH.1995.95GH14
02_AG.GH.2003.03GH184AG
GU201506.1|UniqRecomb|02CM.1807LE|
02_AG.NG.2001.PL0754
BS21
02_AG.GH.1997.97GH17
AD.CI.-.CI32
02_AG.GH.1996.96GH16
02G.GH.1994.94GH09
02_AG.GH.1990.90GH01
02_AG.LR.-.POC44951
43_02G.SA.2003.J11243_118-597
BS47
43_02G.SA.2003.J11223_118-597
BS39
43_02G.SA.2003.J11232_118-597
43_02G.SA.2003.J11456_118-597
02_AG.ES.2006.06SP32_328001
02_AG.CM.2002.02CM_3217MN
02_AG.CM.2001.01CM_0018ND
02_AG.CM.2001.01CM_4410HAL
02_AG.CM.2002.02CM_1677LE
02_AG.CI.-.CI59
02_AG.GA.-.LBV2310
02_AG.CM.2001.01CM_0002BBY
02_AG.GH.2003.03GH181AG
02_AG.GH.1997.97GH23
02_AG.CM.2002.02CM_0014BBY
A1G.CD.1989.VI191_VI1911_ZR_VI191_1847-2091
02_AG.GH.1993.93GH05
02_AG.GH.2003.03GH197AG
02A1.GH.1997.AG2_97GH_AG2_AB052867
02_AG.GW.2004.CC_0030
BS06
02_AG.CM.2001.01CM_0186BA
02_AG.CM.1996.96CM25
02A1.GH.2003.GHNJ176
02D.GH.2003.GHNJ193
BS55_1508-2099
02_AG.GH.2003.GHNJ188
02_AG.US.-.B5512TOB8U
02F.GA.1997.97CIRMF38
02_AG.GH.1997.97GHAG1_AB049811
BS75
BS66
02_AG.GH.1994.94GH10
BS11
02_AG
02_AG.CM.2002.02CM_0015BBY
02_AG.CM.2002.02CM_3217MN_1508-2099
02G.CM.2002.02CM_3228MN
gi|38491595|gb|AY371133.1|_HIV-1_isolate_01CM.0191
02_AG.ES.2007.07SP54_371139
02_AG.GH.2003.03GH182AG
02_AG.CI.-.CI20
02F.GA.1997.97CIRMF35
02_AG.US.2006.BEC146
02_AG.CM.2001.01CM_0126MA
02_AG.CM.2001.01CM_0008BBY
02_AG.CM.2002.02CM_4082STN
02_AG.CM.1997.CM52885
02_AG.CM.1997.97CM21
BS45
BS56
BS38
02_AG.CM.2002.02CM_1901LE
02_AG.CM.2001.01CM_0005BBY
BS16
BS42
02_AG.CM.2002.02CM_3153MN_1508-2099
BS19
BS50
02_AG.CM.1999.pBD6_15
02_AG.CM.2002.02CM_3153MN
gi|38491614|gb|AY371135.1|_HIV-1_isolate_01CM.1188
BS14
BS32
02_AG.ES.2006.06SP7_306121
BS55
02O.CM.1997.97CA_MP645M_O
BS22
BS05_1636-1996
02_AG.CM.2002.02CM_0013BBY
BS64
02_AG.CM.2001.01CM_1475MV
02_AG.CM.2002.02CM_2172SA
02_AG.CM.2002.02CM_2162SA
BS43
BS05
BS71
BS77
02_AG.CM.2001.01CM_0131NY
02_AG.CM.1997.97CM_MP807
0225.CM.-.2931HA
02_AG.CM.2001.01CM_0144ND
02_AG.CM.2001.01CM_0158ND
02_AG.CM.2001.01CM_1237NG
02_AG.CM.2001.01CM_0119MA
BS73
02_AG.CM.2001.01CM_0074NY
BS23
02_AG.CM.2001.01CM_0925MO
BS25
BS09
02_AG.US.2006.502_2696_LH02
02_AG.CY.2009.CY252
02_AG.CM.2002.02CM_2348SA
AF2G.CM.1997.CM52876
02_AG.GA.1997.97CIRMF04
02AG.CM.2002.02CM_3205MN
02D.GH.2003.194AG09
AF2.CM.2002.02CM_3163MN_23-611
02A1U.CM.2002.02CM_2339SA
BS13
02_AG.CM.2001.01CM_0186BA_1508-2099
0209.CI.2001.01IC_PCI127
0209.CI.2001.01IC_17395
0209.CI.1997.97IC_PCI3
19_cpx.CU.1999.CU29_30-531
19_cpx.CU.1999.CU7_30-531
19_cpx, 37_cpx
37_cpx.CM.2000.00CMNYU926
BS24
37_cpx.CM.1997.CM53392
37_cpx.CY.2007.CY192
19_cpx.CU.1999.CU38_30-531
BS27
04_cpx.CY.1994.94CY032_3
04_cpx
04_cpx.CY.2005.CY081
04_cpx.GR.1997.GR84_97PVMY
04_cpx.GR.1991.GR11_97PVCH
A1GU.CD.1976.Z321_Z321B_1602-2105
45_cpx.CD.1997.97CD_MBFE185(2)
45_cpx.CD.1997.97CD_MBFE185
A1.CY.2007.CY185
45_cpx.GA.1997.97GA_TB45
45_cpx.GA.1997.97GA_TB45(2)
45_cpx.CD.1997.97CD_MBS30(2)
45_cpx
45_cpx.CD.1997.97CD_MBS30
A1DK.CD.1985.MAL_MALCG
45_cpx.CM.1997.97CM_MP814(2)
45_cpx.CM.1997.97CM_MP814
DK.GA.1997.97CIRMF15
DK.GA.1997.97CIRMF44
AD.GA.-.G141
DK.GA.1999.99CIRMF01
DK.GA.1996.96CIRMF41
45_cpx.FR.2004.04FR-AUK
02A.GH.1997.G829
09_cpx.SN.1995.95SN7808
09_cpx.SN.1995.95SN1795
09_cpx
0209.CI.2001.01IC_PCI118
09_cpx.GH.1996.96GH2911
09_cpx.GH.1996.96GH2911(2)
09_cpx.CI.2000.00IC_10092
AHJU.CM.2001.01CM_1278NG
AHJU.CM.2001.01CM_1296NG
BS02
32_06A1.EE.2001.EE0369_593-1589
32_06A1.EE.2002.EST2002_1169_593-1589
03_AB.RU.1998.RU98001_98RU001
03_AB.RU.1997.KAL153_2
03_AB.BY.2000.98BY10443
A1.RU.2000.RU00051
01A1.CM.2001.01CM_1152NG
BS10
A.KE.1996.M133gag
A.CD.-.VI57
A1.KE.2002.ML2014_1636-1996
A1.RW.1992.92RW008
A.KE.1998.M264_gag
A1.CA.-.BCCFE_HOMER_HIV_GAG_3062
A1.KE.2006.06KECst_005
A.KE.1998.M428gag
A.KE.1998.M178gag
A.KE.1997.M141gag
A.KE.1997.M21_ps1
A1.KE.1986.ML013_10
A.KE.1997.M104gag
A1.TZ.2003.H594
A.KE.1998.M369gag
C.ZM.2006.ZM1464F
35_AD.AF.2007.221H
35_AD.AF.2006.007H
35_AD.AF.2006.073H
35_AD.AF.2006.077H
35_AD.AF.2007.273H
35_AD.AF.2007.169H
35_AD.AF.2006.047H
A1.KE.2002.ML2014
A.KE.1999.M439gag
A.CI.-.CI4
A1.UG.2005.P0218_130705
A1.GA.-.LBV23
A1H.CM.2001.01CM_4038STN
02A1.GA.1997.97CIRMF58
33_01B.MY.2003.03MYKL010_1
33_01B.MY.2005.05MYKL004_1
33_01B.MY.2004.04MYKL017_1
33_01B.MY.2004.04MYKL008_1
33_01B.MY.2005.05MYKL031_1
33_01B.ID.2007.JKT189-C
33_01B.MY.2005.05MYKL045_1
01_AE
33_01B.MY.2004.04MYKL014_1
33_01B.MY.2004.04MYKL001_1
48_01B.MY.2007.07MYKT016(2)
A1
48_01B.MY.2007.07MYKT016
48_01B.MY.2007.07MYKT021
48_01B.MY.2007.07MYKT014
33_01B.MY.2004.04MYKL013_1
15_01B.TH.2002.02TH_OUR1331
15_01B.TH.2002.02TH_OUR1332
15_01B.TH.1999.99TH_MU2079
15_01B.TH.1999.99TH_R2399
01_AE.CF.1990.90CF11697
01_AE.CF.1990.90CF402_90CR402_CAR_E_4002
01_AE.CF.1990.90CF4071
BS26
22_01A1.ES.2006.06SP42_303556
01A1.CM.2002.02CM_1867LE
01AF2U.CM.2001.01CM_0130NY
AG.CM.2002.02CAMLT04
01A1.CM.2002.02CAMLT72
22_01A1
22_01A1.CM.2002.02CM_3097MN
GQ229529.1|CRF22_01A1|02CM.3097MN|
22_01A1.CM.2001.01CM_0001BBY
BS65
22_01A1.CM.1997.CM53122_1508-2099
22_01A1.CM.1997.CM53122
gi|282598050|gb|GU201503.1|_HIV-1_isolate_01CM.136
0102A.CM.2001.01CM_0190MA
BS40
36_cpx
36_cpx.CM.2000.00CMNYU1162
GU201507.1|UniqRecomb|02CM.1590LE|
36_cpx.CM.2000.00CMNYU830
BS72
A1.ZA.2004.04ZASK162B1
A2.CY.1994.94CY017_AF286242
A2.CY.1994.94CY017_41
A2.CY.1994.CY042GAG_94CY042_2G
A2.CY.1994.CY034G_94CY034_3G
A2.CY.1994.CY049GAG_94CY049_7G
A2.CM.2001.01CM_1445MV
A2.CD.1997.97CDKTB48(2)
A2.CD.1997.97CDKTB48
A2G.CD.1997.97CDKP58
A2.CD.1997.97CDKFE4
A2.KE.1997.clone_78
16_A2D.KR.1997.97KR004
A2
A2.KE.1998.M389_clone17
A2.ZM.2006.32G
A2.KE.2002.clone_139
U.CA.1999.TV721
U.CA.2001.TV749
A.CD.1997.97CD_KCC2(2)
A.CD.1997.97CD_KCC2
A.CD.1997.97CD_KTB13
A.CD.2002.02CD_KTB035
26_AU.CD.2002.02CD_LBTB084
26C.CD.2002.02CD_LBTB032
26C.CD.1997.97CD_KFE267
26_AU.CD.2002.02CD_MBTB047
26_AU.CD.2002.02CD_KS069
26_AU.CD.1997.97CD_KTB119
26C.CD.1997.MBFE250
26CU.CD.1997.97CD_KMST91
18_cpx.CM.1997.CM53379
gi|14290028|gb|AF377959.1|_HIV-1_isolate_CM53379_f
18_cpx
18_cpx.CU.1999.CU76
18_cpx.CU.1999.CU68
18_cpx.CU.1999.CU14
18_cpx.CU.1999.CU68_1508-2099
18_cpx.CM.2001.01CM_1122MO
11_cpx.CM.1996.96CM_4496(2)_1596-2136
11_cpx.CM.1996.96CM_4496
GU201510.1|CRF02/CRF11|02CM.1710LE|_1596-2136
11_cpx.CM.1996.96CM_4496(2)
11_cpx.CM.2001.01CM_0186ND_1596-2136
11_cpx.FR.1999.MP1307_1596-2136
11_cpx.CY.2009.CY259
11_cpx.CM.1999.99CMLB40_1596-2136
11_cpx.FR.1999.MP1298_1596-2136
11_cpx.CM.2002.02CM_4118STN_1636-1996
11_cpx.CM.2001.01CM_4041HAN_1636-1996
11_cpx.CM.1997.MP818_1527-2136
11_cpx
11_cpx.CM.1999.99CMLB40
GU201510.1|CRF02/CRF11|02CM.1710LE|
11_cpx.CM.1995.95CM_1816
11_cpx.CM.2002.02CM_4118STN
11_cpx.CM.2001.01CM_4041HAN
11_cpx.CM.2002.02CM_2190SA_1596-2136
11_cpx.CM.1997.MP818
BS57_1596-2136
11_cpx.FR.1999.MP1307
11_cpx.GR.-.GR17
11_cpx.FR.1999.MP1298
11_cpx.CM.2001.01CM_0186ND
11_cpx.CM.2002.02CM_2190SA
11_cpx.CM.1995.95CM_1816_1596-2136
BS57
49_cpx.GM.2002.N18380
49_cpx.GM.1997.N28353
49_cpx.GM.2003.N26677
49_cpx
A1GHU.GA.-.VI354
U.GR.1999.GR303_99GR303_AY046058
U.GA.1997.97CIRMF02
DU.GA.1997.97CIRMF27
ACFG.GA.1989.LBV105
02GK.CI.2001.01IC_PCI123_963-2105
06_cpx.SN.1997.97SE1078
06_cpx.AU.1996.BFP90
06_cpx.CD.-.BCF-Dioum
06_cpx.CN.2000.00CNLN03
06_cpx.CN.2000.00CNLN03_963-2105
06_cpx.ML.1995.95ML84
06_cpx.ML.1995.95ML127
06_cpx.EE.2001.EE0359_963-2105
06_cpx.RU.2005.04RU001
32_06A1.EE.2001.EE0369
32_06A1.EE.2002.EST2002_1169
06_cpx.EE.2001.EE0359
06_cpx.NG.1997.97NG18
AG.GH.1997.97GH22
06_cpx
06_cpx.GH.2003.03GH173_06
06_cpx.ML.1995.95ML84_963-2105
U.GA.1997.97CIRMF37_1602-2105
06_cpx.CD.-.BCF-Dioum_963-2105
02G.GH.1994.94GH09_1396-1749
06_cpx.AU.1996.BFP90_963-2105
06_cpx.NG.1997.97NG18_963-2105
04_cpx.GR.1997.GR84_97PVMY_1246-1513
06_cpx.ES.2007.07SP47_353742_963-2105
04_cpx.CY.1994.94CY032_3_1246-1513
04_cpx.CY.2005.CY081_1246-1513
04_cpx.GR.1991.GR11_97PVCH_1246-1513
0225.CM.-.2931HA_1225-1573
AG.GH.1997.97GH19
AG.GH.1997.97GH22_963-2105
02GK.CI.2001.01IC_PCI123
06_cpx.ES.2007.07SP47_353742
06_cpx.GH.2003.03GH173_06_963-2105
25_cpx.CM.2006.06CM_BA_040_57-697
25_cpx.CM.2006.06CM_BA_040(2)_57-697
CU.GA.1997.97CIRMF63
25_cpx.SA.2003.J11233_57-697
25_cpx.SA.2003.J11451_57-697
25_cpx
25_cpx.CM.2002.1918LE_57-697
25_cpx.CM.2001.101BA_57-697
25_cpx.CM.2001.101BA_1508-2099
25_cpx.CM.2002.1918LE
25_cpx.CM.2001.101BA
25_cpx.CM.2006.06CM_BA_040(2)
25_cpx.CM.2006.06CM_BA_040
25_cpx.SA.2003.J11233
25_cpx.SA.2003.J11451
25_cpx.SA.2003.J11233_1508-2099
G.RU.2004.Pat16
G.RU.2004.Pat10
G.RU.2006.Pat04
A1G.CD.1989.VI191_VI1911_ZR_VI191
A1GU.CD.1976.Z321_Z321B
14_BG.PT.2000.00PTHSM5
14_BG.PT.2000.00PTHDE10
G.ES.1999.X138_1508-2099
14_BG.ES.2000.X623
14_BG.ES.2000.X477
14_BG.ES.2000.X475
14_BG.ES.2005.X772-8
14_BG.ES.2004.X1589-2_1602-2105
14_BG.ES.1999.X421
14_BG.ES.2000.X605
14_BG.ES.1999.X397
14_BG.ES.2004.X1589-2
G.ES.1999.X138
14_BG.ES.2005.X1870
BS12
G.CM.2001.A1786
24_BG.CU.2003.CB228
24_BG.ES.2008.X2456-2
24_BG.CU.2003.CB219
24_BG.CU.2003.CB340
24_BG.CU.2003.CB619
24_BG.CU.2003.CB570
24_BG.CU.2003.CB378
24_BG.CU.2003.CB621
24_BG.CU.2003.CB471
23_BG.CU.2003.CB118
23_BG.CU.2003.CB347
G
20_BG.CU.1999.Cu103
20_BG.CU.2003.CB134
20_BG.ES.1999.R77
G.CM.1996.96CMABB55(2)
G.CM.1996.96CMABB55
BS03
G.CM.2004.314_40
G.KE.1993.HH8793
G.GH.2003.03GH175G
G.BE.1996.DRCBL
G.CM.2001.01CM_4049HAN
G.CM.2004.944_5
BS04
G.CN.2006.sh52
GU201509.1|CRF02/G|03CM.2848YI|
G.NG.1992.92NG083_JV10832
13_cpx.CM.2002.02CM_3226MN
13_cpx.CM.1996.96CM_1849
13_cpx.CM.2004.04CM_173_9
13_cpx.CM.1996.96CM_4164
13_cpx.CM.2004.04CM_632_28
13_cpx.CM.2004.04CM_632_28(2)
13_cpx.CM.2002.02CM_A1394
G.CD.1991.91ZR02
U.GA.1997.97CIRMF37
G.GA.-.LBV217
A1G.NG.1992.92NG003
G.CM.2004.178_15
G.CN.2002.02CNLN35
BS48
G.CM.2004.515_28_1508-2099
BS46
G.CM.2004.515_28
G.NG.2001.01NGPL0669
G.NG.2001.01NGPL0760
G.NG.2001.PL0567
G.NG.2001.01NGPL0674
43_02G.SA.2003.J11243
43_02G.SA.2003.J11223
43_02G.SA.2003.J11456_1602-2105
43_02G.SA.2003.J11232
43_02G.SA.2003.J11456
AG.NG.2001.PL0209P
BS51
01GHJKU.CD.1997.97CDKFE45
27_cpx.CD.1997.97CDKTB49
27_cpx.FR.2004.04CD_FR_KZS
27_cpx.CD.2002.02CD_LBR024
11_cpx.GR.-.GR17_1596-2136
AGU.CM.2001.01CM_0989MO
AGKU.CD.-.BCF-Kita
U.CY.2008.CY223
U.CD.1983.83CD003_Z3
U.CD.1990.90CD121E12_AF457101
AD.CI.-.CI32_617-1046
02D.GH.2003.194AG09_617-1046
D.FR.-.Vis20
DU.GA.1997.97CIRMF27_182-956
BS30
D.CM.-.CA04
D.TD.1999.MN012
D.CM.2001.01CM_0175BA
D.CM.2001.01CM_0009BBY
D.CM.2001.01CM_4412HAL
BS54
01ADF2.CM.2001.01CM_0908MO
01DU.CM.2001.01CM_4008HAN
DK.GA.1997.97CIRMF15_35-210
AD.GA.-.G141_35-210
DK.GA.1997.97CIRMF44_35-210
D.NL.-.M12020
D.CD.1984.84ZR085
D.CD.1985.Z2Z6_Z2_CDC_Z34
D.CD.1983.ELI
D.CD.1983.NDK
D
D.ZA.1985.R286
16_A2D.KR.1997.97KR004_1-266
05_DF.BE.1993.VI961
05_DF.ES.1999.X492
05_DF.BE.-.VI1310_AF193253
19_cpx.CU.1999.CU7
19_cpx.CU.1999.CU38
D.GA.-.G109
19_cpx.CU.1999.CU29
D.GA.1997.97CIRMF05
A2.KE.1997.clone_78_22-277
D.CD.-.VI203
A2.KE.1998.M389_clone17_22-277
10_CD.TZ.1996.96TZ_BF061
10_CD.TZ.1996.96TZ_BF110
10_CD.TZ.1996.96TZ_BF071
D.KE.1997.ML415_2
D.KE.1998.M389_clone37
02D.GH.2003.194AG09_1047-1222
D.KE.1998.clone_2770
35_AD.AF.2007.169H_1636-1996
A1DK.CD.1985.MAL_MALCG_1508-2099
D.UG.1999.7412_patent
D.UG.1992.92UG001
B.US.-.9P_1189
B.US.1988.WR27(2)
B.CY.2005.CY030_2-704
B.US.2006.26
B.CY.2005.CY030_892-1320
28_BF.BR.1999.BREPM12817_1579-2089
B.DE.-.24
B.GB.-.ChBPt2_A
39_BF.BR.2003.03BRRJ103
B
39_BF.BR.2004.04BRRJ179
39_BF.BR.2003.03BRRJ327
B.AR.1998.1998_44
B.GA.1988.OYI_397
B.JP.1986.JH31
B.FR.1983.HXB2-LAI-IIIB-BRU
B.DE.2004.B_03
B.PE.2006.502_0524_FL06
B.CA.-.BCCFE_HOMER_HIV_GAG_2981
B.CY.2009.CY250
B.ES.2008.X2400-3
B.DK.2004.PMVL_049
35_AD.AF.2006.007H_1508-2099
35_AD.AF.2006.077H_1636-1996
35_AD.AF.2006.073H_1636-1996
35_AD.AF.2007.221H_1636-1996
35_AD.AF.2006.047H_1508-2099
35_AD.AF.2007.273H_1508-2099
F2.CM.2002.02CM_0016BBY
F2.CM.1997.CM53657
AF2G.CM.1997.CM52876_92-786
F2.CM.1995.95CM-MP257
F2
BS49
F2.CM.-.CA20
AF2.CM.2002.02CM_3163MN
F2.CM.-.CA16
01F2.CM.2001.01CM_1402MV
F2.CM.1995.95CM-MP255
BS72_22-642
F1.RO.1996.BCI_R07_908-2101
F1.CD.-.VI174_908-2101
F1.CD.-.VI174
F1.RO.1996.BCI_R07
F1.FI.1993.FIN9363
F1.BR.2006.06BR579
46_BF.BR.1999.99UFRJ_16
12_BF.AR.1997.A32879_AF408629
12_BF.UY.1999.URTR23
12_BF.AR.1997.A32989_AF408630
12_BF.UY.1999.URTR35
12_BF.AR.1989.1989_28
29_BF.BR.2001.BREPM16704_908-2101
28_BF.BR.1999.BREPM12313
40_BF.BR.2005.05BRRJ055
40_BF.BR.2004.04BRRJ115
40_BF.BR.2004.04BRSQ46
40_BF.BR.2005.05BRRJ200
42_BF.LU.2004.luBF_11_04
42_BF.LU.2003.luBF_09_03
42_BF.LU.2005.luBF_17_05
42_BF.LU.2005.luBF_13_05
42_BF.LU.2006.luBF_18_06
42_BF.LU.2003.luBF_04_03
42_BF.LU.2003.luBF_07_03
42_BF.LU.2003.luBF_01_03
42_BF.LU.2003.luBF_02_03
42_BF.LU.2003.luBF_08_03
47_BF.ES.2008.X2457_2
47_BF.ES.2008.P1942
28_BF.BR.1999.BREPM12609
29_BF.BR.1999.BREPM11948
28_BF.BR.1999.BREPM12313_908-2101
29_BF.BR.1999.99UFRJ_1
29_BF.BR.2001.BREPM16704
28_BF.BR.1999.BREPM12609_908-2101
28_BF.BR.1999.BREPM12817
F1.BR.2006.06BR579_908-2101
12_BF.AR.1988.1988_51
12_BF.AR.1999.ARMA159
F1.BR.1993.93BR020_1
46_BF.BR.2001.01BR125_908-2101
46_BF.BR.2007.07BR_FPS742_908-2101
46_BF.BR.2001.01BR087_908-2101
F1
46_BF.BR.2007.07BR_FPS625_908-2101
46_BF.BR.2007.07BR_FPS810_908-2101
46_BF.BR.2007.07BR_FPS783_908-2101
46_BF.BR.2007.07BR_FPS812_908-2101
F1.AR.1995.1995_34
F1.AR.2002.2002_20
47_BF.ES.2008.X2457_2_908-2101
47_BF.ES.2008.P1942_908-2101
40_BF.BR.2005.05BRRJ055_908-2101
40_BF.BR.2004.04BRSQ46_908-2101
40_BF.BR.2004.04BRRJ115_908-2101
40_BF.BR.2005.05BRRJ200_908-2101
12_BF.AR.1997.A32879_AF408629_908-2101
12_BF.AR.1997.A32989_AF408630_908-2101
12_BF.AR.1988.1988_51_908-2101
12_BF.AR.1989.1989_28_908-2101
12_BF.AR.1999.ARMA159_908-2101
F1.AR.1995.1995_34_908-2101
46_BF.BR.2001.01BR087
46_BF.BR.2007.07BR_FPS810
46_BF.BR.2007.07BR_FPS625
46_BF.BR.2007.07BR_FPS783
46_BF.BR.2007.07BR_FPS742
46_BF.BR.2007.07BR_FPS812
44_BF.CL.2000.CH80_908-2101
44_BF.CL.2001.CH12
44_BF.CL.2000.CH80
F1.AR.2002.2002_20_908-2101
46_BF.BR.1999.99UFRJ_16_34-807
F1.AR.2005.2005_08
12_BF.UY.1999.URTR23_908-2101
12_BF.UY.1999.URTR35_908-2101
F1.AR.2005.2005_12
F1.AR.2002.2002_01
46_BF.BR.2001.01BR125
F1.BR.1993.93BR020_1_34-807
42_BF.LU.2003.luBF_08_03_908-2101
42_BF.LU.2006.luBF_18_06_908-2101
42_BF.LU.2003.luBF_07_03_908-2101
42_BF.LU.2005.luBF_17_05_908-2101
42_BF.LU.2005.luBF_13_05_908-2101
42_BF.LU.2003.luBF_04_03_908-2101
42_BF.LU.2003.luBF_02_03_908-2101
42_BF.LU.2003.luBF_01_03_908-2101
42_BF.LU.2004.luBF_11_04_908-2101
42_BF.LU.2003.luBF_09_03_908-2101
F1.FI.1993.FIN9363_908-2101
F1.RU.2008.D88_845
K.CM.1996.96CM-MP535
K.CD.1997.97ZR-EQTB11
U.NL.1995.U_NL_95_H10986_B6
U.NL.1995.U_NL_95_H10986_D1
U.NL.2001.U_NL_01_H10986_D1
U.NL.2001.U_NL_01_H10986_C11
U.CY.2005.CY090
C.ZA.1998.DU151
C.ZA.2006.SK371
08_BC.CN.2002.02CNKM201
08_BC.CN.2001.01CNKM017
08_BC.CN.2000.HH065
08_BC.CN.2000.HH025
08_BC.CN.2000.WS031
08_BC.CN.2000.WS030
08_BC.CN.2006.nx2
08_BC.CN.2005.05LN093
08_BC.CN.2000.00CNLN07
08_BC.CN.2004.04CNLN74
07_BC.CN.2002.02CNLN43
07_BC.CN.2002.02CNLN23
33_01B.MY.2004.04MYKL014_1_1686-2136
07_BC.CN.2001.01CNKM018
33_01B.MY.2004.04MYKL017_1_1686-2136
48_01B.MY.2007.07MYKT016(2)_1686-2136
48_01B.MY.2007.07MYKT016_1686-2136
48_01B.MY.2007.07MYKT014_1686-2136
48_01B.MY.2007.07MYKT021_1686-2136
33_01B.MY.2005.05MYKL031_1_1686-2136
C
33_01B.MY.2004.04MYKL008_1_1686-2136
33_01B.MY.2004.04MYKL001_1_1686-2136
33_01B.MY.2003.03MYKL010_1_1686-2136
33_01B.MY.2005.05MYKL045_1_1686-2136
07_BC.CN.2004.04CNLN79
33_01B.ID.2007.JKT189-C_1686-2136
33_01B.MY.2005.05MYKL004_1_1686-2136
07_BC.CN.2007.07LN134
33_01B.MY.2004.04MYKL013_1_1686-2136
07_BC.CN.1997.97CN001_C54
07_BC.CN.1997.97CNKM007
07_BC.CN.2002.02CNLN41
07_BC.TW.2004.TW_D3
07_BC.CN.2000.WS001
C.IN.1993.93IN904
C.BW.2000.00BW147127
C.TZ.2003.H195
31_BC.BR.2004.04BR142
31_BC.BR.2004.04BR137
31_BC.BR.2002.110PA
C.CD.-.VI313
C.KE.1987.clone_343
C.DJ.-.DJ259
C.TZ.2003.H001
C.ZM.2005.ZM1033M
U.ZM.2003.ZM248F
J.SE.1993.SE9280_7887
J
J.SE.1994.SE9173_7022
J.CM.2004.04CMU11421
J.CD.1997.J_97DC_KTB147
H.CF.1990.056
H.BE.1993.VI997
H
GH.GA.1990.VI525_VI5251
H.GB.2000.00GBAC4001
H.BE.1993.VI991
H.CD.1991.VI557
B.US.-.J7511TOB8U
CPZ.CM.2005.SIVcpzLB7
CPZ
CPZ.CM.-.SIVcpzMB897
CPZ.CM.2005.SIVcpzMB66
N.CM.2006.U14296
N.CM.2002.DJO0131
N.CM.1995.YBF30
N
N.CM.2006.U14842
N.CM.2002.SJGddd
N.CM.2004.04CM_1015_04(2)
N.CM.2004.04CM_1015_04
N.CM.2004.04CM_1131_03
N.CM.1997.YBF106
CPZ.CM.2001.SIVcpzCAM13
P
P.FR.2006.RBF168
P.FR.2009.RBF168
P.CM.2006.U14788(2)
P.CM.2006.U14788
O.CM.1998.98CMABB212
O.CM.1991.MVP5180
O.CM.1994.BCF06
O
O.BE.1987.ANT70
O.CM.-.pCMO2_5
O.US.1997.97US08692A
O.CM.-.pCMO2_3
O.SN.1998.SE42HALD
O.FR.1992.VAU
O.CM.1996.96CMABB009
0.1
