# Supplementary material for: Characterization of HIV-1 gag and nef in Cameroon: further evidence of extreme diversity at the origin of the HIV-1 group M epidemic
Source: Virol J. 2013 Jan 22;10:29. doi: 10.1186/1743-422X-10-29 (PMC3560183; doi:10.1186/1743-422X-10-29)
Supplement: Additional file 2 — Detailed phylogenetic analysis of nucleotide sequences in the nef gene. Maximum likelihood tree indicating the phylogenetic relationships between 628 nef sequences including all sequence identifiers. Blue arrows indicate the outlier sequences found in this study while the green arrows indicate the outlier sequences from previously-characterized Cameroonian sequences. Black squares at the end of the branches represent the nef sequences sampled from Cameroon in this study, while red squares represent intragene recombinant fragments in our samples. [file 1743-422X-10-29-S2.pptx]

## Slide 1
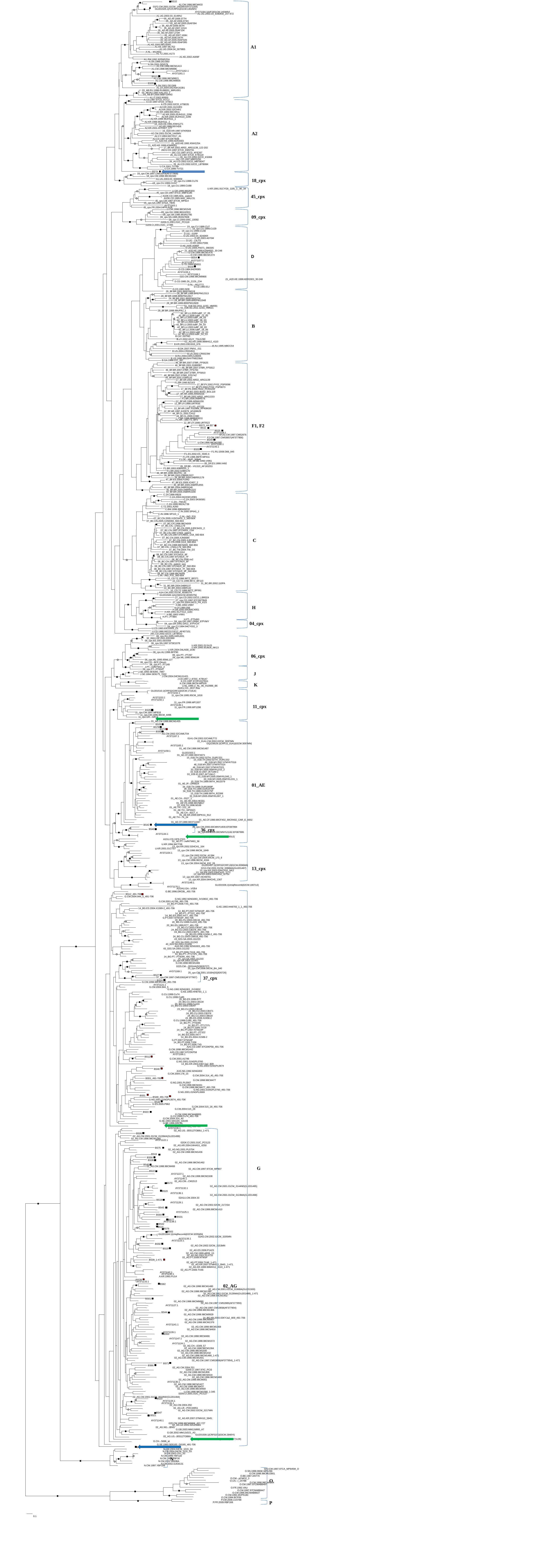

BS10
A1.CM.1998.98CM433
01F2.CM.2001.01CM_1402MV(AY371164)
AY371164.1|A/F2|01CM.1404MV|
GU201505.1|F2/CRF01|01CM.1402MV|
A1.UG.2003.03_9390KM_247-472
A1.UG.2003.03_6148NJ
35_AD.AF.2006.077H
35_AD.AF.2006.073H
35_AD.AF.2005.05AF094
35_AD.AF.2006.007H
35_AD.AF.2007.221H
35_AD.AF.2005.05AF104
35_AD.AF.2007.273H
35_AD.AF.2007.169H
35_AD.AF.2006.047H
35_AD.AF.2005.05AF026
35_AD.AF.2005.05AF095
A1
A1.KE.2000.NKU3005
A1.KE.1997.ML752
A1.UG.2004.04_0079BS
A.NL.-.M12653
A1.TZ.2001.A173
A1.KE.2002.A009F
A1.RW.1992.92RW025A
A.SN.1996.DDJ360
A.SN.2001.DDI579
A1.CM.1998.98CM1413
A1.CM.1998.98CM9896
AY371162.1
AY371161.1
BS13
A1.CM.1998.98CM9821
A1.CM.1998.98CM9835
BS09
A.SN.2001.DDJ369
A1.ZA.2004.04ZASK162B1
03_AB.RU.1998.RU98001_98RU001
03_AB.RU.1997.KAL153_2
03_AB.BY.2000.98BY10443
A1.IT.2002.60000
A.CD.1997.97CD_KCC2
A.CD.1997.97CD_KTB13
A.CD.2002.02CD_KTB035
A2.KR.2001.01CHR3
A2.KR.2002.02CHR1
A2.KR.1999.99CHR11
A2.KR.2005.05JHS10_2296
A2.KR.2005.05JHS10_2295
A2.KR.1999.99JHS11_1
A2.KR.1999.99JHS11_2
16_A2D.KE.1991.KNH1271
A2.KR.1999.99YHD8
A2.KR.2001.01YHD7_177
A2
16_A2D.KR.1997.97KR004
A2.CM.2001.01CM_1445MV
A2.CY.1994.94CY017_41
A2.CD.1997.97CDKTB48
21_A2D.KE.1999.KER2003
21_A2D.KE.1991.KNH1254
21_A2D.KE.1999.KSM4001
17_BF.AR.2002.AR02_ARG1139_122-202
26CU.CD.1997.97CD_KMST91
26C.CD.1997.97CD_KFE267
26_AU.CD.1997.97CD_KTB119
26_AU.CD.2002.02CD_KS069
26C.CD.1997.MBFE250
26_AU.CD.2002.02CD_MBTB047
26_AU.CD.2002.02CD_LBTB084
U.CA.2001.TV749
U.CA.1999.TV721
BS74
18_cpx.CM.1997.CM53379(AF377959)
18_cpx.CM.1998.98CM1583
18_cpx
A1.UG.2003.03_9390KM
18_cpx.CU.1999.CU76
18_cpx.CU.1999.CU14
18_cpx.CU.1999.CU68
U.KR.1991.91CYG8_1185_1:_90_34
U.GR.1999.99GR303
45_cpx.CD.1997.97CD_MBFE185
45_cpx
A1DK.CD.1985.MAL_patent
A1DK.CD.1985.MAL_MALCG
45_cpx.CM.1997.97CM_MP814
45_cpx.GA.1997.97GA_TB45
AY371163.1
45_cpx.FR.2004.04FR-AUK
U.CM.1998.98CM1549
09_cpx.GH.1996.96GH2911
09_cpx
09_cpx.SN.1995.95SN1795
09_cpx.SN.1995.95SN7808
09_cpx.CI.2000.00IC_10092
0209.CI.2001.01IC_PCI118
0209.CI.2001.01IC_17395
19_cpx.CU.1999.CU7
19_cpx.CU.1999.CU29
19_cpx.CU.1999.CU38
D.UG.-.01RP
D.UG.2003.03_9240KR
D.KE.2003.A070M
D.UG.-.13LTS
D.KR.1994.P306
D.KE.2005.A086F
D.UG.2005.P0071_090305
21_A2D.KE.1999.KSM4001_30-248
D.CM.1998.98CM1476
D
D.CM.1998.98CM1374
BS54
AY371157.1
AY371156.1
D.TD.1999.MN011
BS30
D.CD.1984.84ZR085
AY371155.1
AY371168.1
02D.CM.1998.98CM9868
21_A2D.KE.1999.KER2003_30-248
D.CD.1985.Z6_Z2Z6_Z34
D.NL.-.M12772
D.CD.1983.ELI
D.CD.1983.NDK
29_BF.BR.2002.BREPM119
28_BF.BR.1999.BREPM12313
28_BF.BR.1999.BREPM12817
29_BF.BR.2001.BREPM16704
29_BF.BR.1999.BREPM11948
28_BF.BR.1999.BREPM12609
51_01B.SG.2011.11SG_HM091
51_01B.SG.2011.11SG_HM021
29_BF.BR.1999.99UFRJ_1
42_BF.LU.2005.luBF_17_05
42_BF.LU.2005.luBF_13_05
42_BF.LU.2003.luBF_09_03
42_BF.LU.2003.luBF_06_03
42_BF.LU.2004.luBF_11_04
B
42_BF.LU.2003.luBF_04_03
42_BF.LU.2003.luBF_08_03
42_BF.LU.2006.luBF_18_06
42_BF.LU.2003.luBF_01_03
42_BF.LU.2003.luBF_02_03
B.CA.-.007ND
B.UY.2002.02UY_TSU1290
02_AG.KR.1999.99MHI12_4110
B.KR.2003.03GSG5_676
B.AU.1995.MBCC54
B.DK.2007.PMVL_011
B.US.2004.CR0345Q
B.US.2002.CR0023W
B.RU.2004.04RU128005
B.US.1998.98USHVTN8229c6
B.GA.1988.OYI_397
46_BF.BR.2007.07BR_FPS625
46_BF.BR.2001.01BR087
46_BF.BR.2007.07BR_FPS812
46_BF.BR.2007.07BR_FPS783
46_BF.BR.2007.07BR_FPS810
46_BF.BR.2007.07BR_FPS742
46_BF.BR.2001.01BR125
17_BF.AR.2002.AR02_ARG1139
F1.BR.1990.BZ163
17_BF.PY.2002.PY02_PSP0096
17_BF.PY.2002.PY02_PSP0073
17_BF.PE.2002.PE02_PCR0155
17_BF.BO.2002.BO02_BOL119
17_BF.AR.1999.ARMA038
17_BF.AR.2002.AR02_ARG2233
F1.BR.2006.06BR579
12_BF.AR.1999.ARMA159
12_BF.UY.1999.URTR35
F1.PT.-.PT225
12_BF.AR.1997.A32989_AF408630
12_BF.AR.1997.A32879_AF408629
44_BF.CL.2001.CH12
44_BF.CL.2000.CH80
F.BR.1998.98BRRS011
F1.AR.-.181771_BF5
12_BF.UY.1999.URTR23
F1, F2
BS72_44-357
BS11
BS25
AY371158.1
AF2G.CM.1997.CM52876
F2.CM.1997.CM53657(AF377956)
BS49
U.CM.1998.98CM1580
AY371160.1
AY371143.1
BS55
F1.RU.2008.D88_845
F1.ES.2002.ES_X845-4
F1.FR.1996.96FR-MP411
F1.DE.-.MVP_30846
05_DF.BE.1993.VI961
05_DF.ES.1999.X492
05_DF.BE.-.VI1310_AF193253
F1.BR.1993.93BR020_1
F1.BR.2002.02BR170
46_BF.BR.1999.99UFRJ_16
39_BF.BR.2003.03BRRJ327
39_BF.BR.2004.04BRRJ179
47_BF.ES.2008.P1942
47_BF.ES.2008.X2457_2
40_BF.BR.2005.05BRRJ055
40_BF.BR.2004.04BRSQ46
40_BF.BR.2004.04BRRJ115
40_BF.BR.2005.05BRRJ200
C.ZA.1999.RB28
C.ZA.2004.04ZASK145B1
C.ZA.2003.SK065B1
C.ZA.-.TM14C
C.ZA.1999.99ZALT39
C.TZ.2001.A260
C.BW.1996.96BWM032
C.IN.2000.SP641_1
C.IN.1996.SP110_1
C.IN.-.IND_P31
07_BC.CN.2005.XJDC6431_2_560-804
07_BC.CN.2005.XJN0084_560-804
07_BC.CN.1998.98CN009
07_BC.CN.-.CNGL179
07_BC.CN.2005.XJDC6431_2
07_BC.CN.1997.97CN001_C54
07_BC.CN.1997.CN54_patent
07_BC.CN.1997.97CN001_C54_560-804
C
07_BC.CN.2005.XJN0084
07_BC.CN.2005.XJDC6441
07_BC.CN.2008.1114_560-804
07_BC.CN.1998.98CN009_560-804
07_BC.CN.-.CNGL179_560-804
07_BC.TW.2004.TW_D3
07_BC.CN.2008.1114
08_BC.CN.1997.97CNGX_9F
08_BC.CN.1997.97CNGX_7F
08_BC.CN.2006.nx2
08_BC.CN.1997.97CNGX_6F
08_BC.CN.-.patent_seq
08_BC.CN.1997.97CNGX_6F_560-804
08_BC.CN.1997.97CNGX_7F_560-804
08_BC.CN.1997.97CNGX_9F_560-804
08_BC.CN.1998.98CN006
C.IN.-.IND_P31_560-804
10_CD.TZ.1996.96TZ_BF071
10_CD.TZ.1996.96TZ_BF110
31_BC.BR.2002.110PA
31_BC.BR.2004.04BR137
31_BC.BR.2004.04BR142
10_CD.TZ.1996.96TZ_BF061
A1H.CM.2001.01CM_4038STN
GU201508.1|A1/H|01CM.4038STN|
27_cpx.CD.2002.02CD_LBR024
27_cpx.CD.1997.97CDKTB49
27_cpx.FR.2004.04CD_FR_KZS
H
H.BE.1993.VI997
H.CF.1990.056
H.GB.2000.00GBAC4001
H.KR.1991.91JTS12_1183
H.BE.1993.VI991
H.PT.-.PT964
H.PT.-.PT5493
04_cpx
04_cpx.GR.1997.GR84_97PVMY
04_cpx.GR.1991.GR11_97PVCH
04_cpx.CY.1994.94CY032_3
U.CD.1983.83CD003_Z3
U.CD.1990.90CD121E12_AF457101
26C.CD.2002.02CD_LBTB032
06_cpx.RU.2005.04RU001
32_06A1.EE.2001.EE0369
06_cpx.EE.2001.EE0359
06_cpx.SN.1997.97SE1078
U.KR.2001.01YHJ3
U.KR.1993.93JBJ6_NK13
U.KR.2004.04LNS8_1540
06_cpx.AU.1996.BFP90
06_cpx
06_cpx.PT.-.PT297
06_cpx.ML.1995.95ML84
06_cpx.ML.1995.95ML127
06_cpx.CD.-.BCF-Dioum
06_cpx.PT.-.PT149
J.PT.-.nefNTM11_2
06_cpx.PT.-.PT6047
J
J.SE.1993.SE9280_7887
J.SE.1994.SE9173_7022
J.CM.2004.04CMU11421
J.CD.1997.J_97DC_KTB147
K.CD.1997.97ZR-EQTB11
K
K.CM.1996.96CM-MP535
U.NL.1995.U_NL_95_H10986_B6
AGKU.CD.-.BCF-Kita
GU201510.1|CRF02/CRF11|02CM.1710LE|
AY371152.1
11_cpx.CM.1995.95CM_1816
AY371153.1
AY371150.1
11_cpx.FR.1999.MP1307
11_cpx
AY371149.1
11_cpx.FR.1999.MP1298
BS35
11_cpx.CM.1997.MP818
11_cpx.CM.1996.96CM_4496
11_cpx.GR.-.GR17
AY371151.1
01_AE.CM.1998.98CM1435
BS26
BS78
BS72
BS65
AG.CM.2002.02CAMLT04
AY371167.1
01A1.CM.2002.02CAMLT72
22_01A1.CM.2002.02CM_3097MN
GQ229529.1|CRF22_01A1|02CM.3097MN|
AY371165.1
01_AE.CM.1998.98CM1487
AY371159.1
GU201503.1
01_AE.CF.1990.90CF4071
15_01B.TH.2002.02TH_OUR1331
15_01B.TH.2002.02TH_OUR1332
48_01B.MY.2007.07MYKT016
48_01B.MY.2007.07MYKT014
48_01B.MY.2007.07MYKT021
33_01B.MY.2005.05MYKL015_2
33_01B.ID.2007.JKT189-C
33_01B.ID.2007.JKT194-C
33_01B.MY.2005.05MYKL045_1
33_01B.MY.2005.05MYKL031_1
15_01B.TH.1999.99TH_MU2079
01_AE
01_AE.JP.-.DR6824
34_01B.TH.1999.OUR1969P
34_01B.TH.1999.OUR2478P
34_01B.TH.1999.OUR2275P
15_01B.TH.1999.99TH_R2399
33_01B.MY.2005.05MYKL007_1
01_AE.CH.-.5527_1
01_AE.HK.2004.HK001
01_AE.VN.1998.98VNBG7
15_01B.TH.1996.M169
01_AE.TH.-.122_16
01_AE.TH.-.NP03(2)
01_AE.CH.-.6017_4
01_AE.KR.2000.00PKI11_812
01_AE.TH.-.74_24
01_AE.CF.1990.90CF402_90CR402_CAR_E_4002
01_AE.CF.1990.90CF11697
BS42
36_cpx.CM.2000.00CMNYU830.EF087994
36_cpx
BS40
36_cpx.CM.2000.00CMNYU1162.EF087995
AY371144.1
GU201507.1|UniqRecomb|02CM.1590LE|
A1GU.CD.1976.Z321
01_AE.PT.-.nefNTM63_56
U.KR.1994.94CTS9
13_cpx.KR.2002.02HCH1_164
U.KR.2001.01CTS7
13_cpx.CM.1996.96CM_1849
AY371154.1
13_cpx.CM.2002.02CM_A1394
13_cpx.CM.2004.04CM_173_9
13_cpx.CM.1996.96CM_4164
13_cpx.CM.2004.04CM_632_28
GU201497.1|CRF02/CRF13|01CM.0096MA|
13_cpx
0213.CM.2001.01CM_0096MA(GU201497)
13_cpx.KR.2003.03HCH10_NK3
13_cpx.KR.2003.03HCH10_1172
13_cpx.KR.2003.03HCH10_1172s
13_cpx.KR.1997.HCH9701
13_cpx.KR.2004.04HCH5_1367
AY371148.1
GU201506.1|UniqRecomb|02CM.1807LE|
AY371170.1
A1GHU.GA.-.VI354
G.BE.1996.DRCBL_491-706
BS12_491-706
G.CM.2004.944_5_491-706
G.NG.1992.92NG083_JV10832_491-706
G.CM.2001.A1786_491-706
14_BG.PT.2006.TX5_491-706
G.KE.1993.HH8793_1_1_491-706
14_BG.ES.2004.X1589-2_491-706
14_BG.PT.2007.NTM10P_491-706
14_BG.PT.-.PT322_491-706
14_BG.ES.2000.X477_491-706
G.PT.2007.NTM16P_491-706
20_BG.CU.2003.CB134_491-706
20_BG.CU.1999.Cu103_491-706
20_BG.ES.1999.R77_491-706
23_BG.CU.2003.CB347_491-706
24_BG.CU.2003.CB378_491-706
24_BG.CU.2003.CB471_491-706
24_BG.ES.2008.X2456-2_491-706
24_BG.CU.2003.CB619_491-706
43_02G.SA.2003.J11223
43_02G.SA.2003.J11243
43_02G.SA.2003.J11456
A1G.NG.1992.92NG003_491-706
43_02G.SA.2003.J11232
14_BG.PT.2006.TX19_491-706
14_BG.PT.-.PT1737c_491-706
14_BG.PT.-.PT9295_491-706
25_cpx.SA.2003.J11233
25_cpx.SA.2003.J11451
G.CM.1998.98CM1466
0225.CM.-.2931HA(DQ826727)
25_cpx.CM.2006.06CM_BA_040
AY371169.1
25_cpx.CM.2001.101BA(DQ826726)
BS27
37_cpx
37_cpx.CM.1997.CM53392(AF377957)
BS24
G.CM.1998.98CM1442_491-706
AY371121.1
G.CM.2004.944_5
G.NG.1992.92NG083_JV10832
G.KE.1993.HH8793_1_1
G.CU.1999.Cu74
G.CU.1999.Cu85
20_BG.ES.1999.R77
20_BG.CU.2003.CB134
20_BG.CU.1999.Cu103
23_BG.CU.2003.CB347
23_BG.CU.2003.CB118
24_BG.CU.2003.CB471
24_BG.CU.2003.CB378
24_BG.CU.2003.CB619
24_BG.ES.2008.X2456-2
G.CU.1999.Cu85_491-706
14_BG.PT.-.PT9295
14_BG.PT.-.PT1737c
14_BG.PT.2006.TX19
14_BG.PT.2007.NTM10P
14_BG.PT.-.PT322
14_BG.ES.2000.X477
14_BG.ES.2004.X1589-2
G.PT.2007.NTM16P
14_BG.PT.2006.TX56
14_BG.PT.2006.TX5
A2G.CD.1997.97CDKP58_491-706
G.CM.1998.98CM1442
A2G.CD.1997.97CDKP58
AY371166.1
BS12
G.CM.2001.A1786
G.NG.2001.01NGPL0760
14_BG.KR.2003.03KYJa2_809
G.NG.2001.01NGPL0674
BS48
A1G.NG.1992.92NG003
G.CM.2004.178_15
G.CM.2004.314_40_491-706
BS51_491-706
G.CM.1998.98CM477
G.NG.2001.PL0567
G.CM.1998.98CM431
G.CM.1998.98CM477_491-706
G.NG.2001.01NGPL0760_491-706
G.NG.2001.01NGPL0669
BS51
BS48_491-706
G.NG.2001.01NGPL0674_491-706
BS46
G.ES.2005.P962
G.CM.2004.515_28_491-706
G.CM.2004.515_28
BS03
G.CM.1996.96CMABB55
G.CU.1999.Cu74_491-706
G.CM.2004.314_40
G.SE.1993.SE6165_G6165
G.BE.1996.DRCBL
G.CM.1998.98CM431_491-706
AY371126.1
02_AG.US.-.B5512TOB8U_1-471
BS06
02_AG.CM.2001.01CM_0126MA(GU201499)
02_AG.CM.1998.98CM1360
AY371122.1
02GK.CI.2001.01IC_PCI123
02_AG.KR.2004.04HAS1_4250
BS75
02_AG.NG.2001.PL0754
02_AG.CM.1998.98CM1436
BS16
BS56
BS38
02_AG.CM.1998.98CM1462
BS45
G
02_AG.CM.1998.98CM468
02_AG.CM.1997.97CM_MP807
BS22
AY371137.1
02_AG.CM.1998.98CM1508
AY371145.1
02_AG.CM.-.CM1513
BS73
02_AG.CM.2001.01CM_0144ND(GU201495)
AY371132.1
BS20
AY371136.1
02_AG.CM.2001.01CM_0119MA(GU201498)
02A1U.CM.2004.33
BS18
AY371129.1
02_AG.CM.2002.02CM_2172SA
BS43
02_AG.CM.1998.98CM1410
AY371125.1
BS64
BS31
BS77
AY371138.1
BS32
BS14
BS79
BS02
GU201504.1|UniqRecomb|02CM.3205MN|
02AG.CM.2002.02CM_3205MN
AY371135.1
AY371123.1
BS50
02_AG.CM.2002.02CM_3153MN
BS19
02_AG.ES.2006.P1423
02_AG.CM.1999.pBD6_15
02_AG.NG.2001.PL0710
02_AG.PT.2008.NTM3P
BS39_1-471
02_AG.PT.2006.TX46_1-471
02_AG.KR.2007.07MHI10_3945:_1-471
02_AG.KR.1999.99MHI12_4110_1-471
02_AG.PT.2006.TX46
AY371142.1
AY371140.1
A.KR.1993.PU14
BS39
AY371133.1
02_AG
BS82
02_AG.CM.1998.98CM1480
02_AG.CM.2001.01CM_0186BA(GU201500)
02_AG.CM.1998.98CM1390
02_AG.CM.2001.01CM_0126MA(GU201499)_1-471
02_AG.CM.1998.98CM1452
BS01
02_AG.CM.1998.98CM9890
02_AG.CM.1997.CM52885(AF377955)
AY371127.1
02_AG.CM.1997.CM53658(AF377954)
02_AG.CM.1998.98CM1366
BS44
02_AG.CM.1998.98CM9929
14_BG.KR.2003.03KYJa2_809_491-706
02_AG.CM.1998.98CM9907
02_AG.CM.1998.98CM1376
AY371141.1
02_AG.CM.1998.98CM1468
02_AG.CM.1998.98CM459
AY371139.1
BS53
02_AG.CM.1998.98CM466
AY371147.1
02_AG.CM.1998.98CM1572
AY371124.1
02_AG.CH.-.6349_57
02_AG.CM.1998.98CM1364
02_AG.CM.1998.98CM1500
02_AG.CM.1998.98CM1432
02_AG.CM.1998.98CM1488_1-471
02_AG.CM.1998.98CM1401
02_AG.CM.1997.CM53658(AF377954)_1-471
BS71
BS66
02_AG.CM.2004.251
0209.CI.1997.97IC_PCI3
02_AG.CM.1998.98CM1409
02_AG.CM.1998.98CM419
02_AG.CM.1998.98CM1488
02_AG.CM.1998.98CM001
AY371130.1
02_AG.CM.1998.98CM1427
02_AG.CM.1998.98CM437
02_AG.CM.1998.98CM469
U.CM.1998.98CM1580_1-345
0209.CI.2001.01IC_PCI127
02_AG.CM.2001.01CM_0018ND(GU201494)
BS23
AY371134.1
AY371131.1
02_AG.CM.2004.250
02_AG.LR.-.POC44951
02_AG.CM.2002.02CM_3217MN
BS47
BS21
02_AG.KR.2007.07MHI10_3945:
AY371146.1
02D.CM.1998.98CM9868_457-727
02_AG.UZ.2002.02UZ0683
02_AG.NG.-.IBNG
G.GB.2003.MM12d900_A7
G.GB.2002.MM12d321_A3
GU201509.1|CRF02/G|03CM.2848YI|
02_AG.US.-.B5512TOB8U
02_AG.CM.2002.02CM_1970LE(AY371128)
G.CH.-.5696_at
G.SE.1993.SE6165_G6165_491-706
BS29
N.CM.2004.04CM_1015_04
N.CM.2004.04CM_1131_03
N.CM.2002.CK1_62
N.CM.1999.YBF116
N
N.CM.1995.YBF30
N.CM.2001.2693BA
N.CM.2002.DJO0131
N.CM.1997.YBF106
02O.CM.1997.97CA_MP645M_O
O.SN.1999.99SE-MP1299
O.CM.1998.98CMU2901
O.BE.1987.ANT70
O
O.CM.-.pCMO2_3
O.US.-.I_2478B
O.CM.1998.98CMA104
O.CM.1997.97CMABB497
O.FR.1992.VAU
O.CM.1997.97CMABB447
O.CM.1996.96CMABB637
O.CM.1991.MVP5180
O.CM.1994.BCF06
P.CM.2006.U14788
P
P.FR.2009.RBF168
0.1
